# Enhanced Enzymatic Activity of Glycerol-3-Phosphate Dehydrogenase from the Cryophilic *Saccharomyces kudriavzevii*

**DOI:** 10.1371/journal.pone.0087290

**Published:** 2014-01-30

**Authors:** Bruno M. Oliveira, Eladio Barrio, Amparo Querol, Roberto Pérez-Torrado

**Affiliations:** 1 Instituto de Agroquímica y Tecnología de los Alimentos, IATA-CSIC, E-46980, Paterna (Valencia), Spain; 2 Institut “Cavanilles” de Biodiversitat i Biologia Evolutiva, Universitat de València, València, Spain; University Paris South, France

## Abstract

During the evolution of the different species classified within the *Saccharomyces* genus, each one has adapted to live in different environments. One of the most important parameters that have influenced the evolution of *Saccharomyces* species is the temperature. Here we have focused on the study of the ability of certain species as *Saccharomyces kudriavzevii* to grow at low temperatures, in contrast to *Saccharomyces cerevisiae*. We observed that *S. kudriavzevii* strains isolated from several regions are able to synthesize higher amounts of glycerol, a molecule that has been shown to accumulate in response to freeze and cold stress. To explain this observation at the molecular level we studied the expression of glycerol biosynthetic pathway genes and we observed a higher expression of *GPD1* gene in *S. kudriavzevii* compared to *S. cerevisiae* in micro-vinification conditions. We observed higher enzymatic activity of Gpd1p in *S. kudriavzevii* in response to osmotic and cold stress. Also, we determined that *S. kudriavzevii* Gpd1p enzyme presents increased catalytic properties that will contribute to increase glycerol production. Finally, we evaluated the glycerol production with *S. cerevisiae, S. kudriavzevii* or a recombinant Gpd1p variant in the same background and observed that the *S. kudriavzevii* enzyme produced increased glycerol levels at 12 or 28°C. This suggests that glycerol is increased in *S. kudriavzevii* mainly due to increased *V*_max_ of the Gpd1p enzyme. All these differences indicate that *S. kudriavzevii* has changed the metabolism to promote the branch of the glycolytic pathway involved in glycerol production to adapt to low temperature environments and maintain the NAD^+^/NADH ratio in alcoholic fermentations. This knowledge is industrially relevant due to the potential use, for example, of *S. cerevisiae*-*S. kudriavzevii* hybrids in the wine industry where glycerol content is an important quality parameter.

## Introduction

*Saccharomyces kudriavzevii* is a species classified within the *Saccharomyces* genus, which is phylogenetically closely related to *Saccharomyces cerevisiae*. The similarity between these two species prompted the formation of natural interspecific hybrids, present in wine [1] and brewing environments [2]. Initially, a few strains of *S. kudriavzevii* were described isolated in decayed leaves and soil in Japan [3] but recently, several Iberian populations have been described [4], [5]. In fact, these isolation events were made possible due to the decrease in isolation temperatures, revealing one of the most interesting characteristics of *S. kudriavzevii* species, its adaptation to low temperature. Previous works have shown *S. kudriavzevii* to outperform *S. cerevisiae* strains in different low temperature conditions in natural grape juice fermentations [6] or synthetic media growth [7], [8]. Another important difference between the two species is that *S. kudriavzevii* produces higher amounts of glycerol during low temperature fermentations than *S. cerevisiae* [1].

Several features have been related to low temperature adaptation of yeasts, including membrane lipid composition, synthesis of ribosomal proteins and trehalose content [9]. More recently, evidence has been found of the role played by glycerol production in cold stress [10], via a regulatory mechanism involving the HOG (High Osmolarity Glycerol) pathway [11], [12]. Intracellular glycerol content was linked to the *S. cerevisiae* cell survival in fermentations after freezing and at low temperatures [10]. In this respect, intracellular glycerol accumulation was observed in response to cold stress, indicating the involvement of this molecule when cells face low or near freezing temperature conditions. Furthermore, intracellular glycerol is also involved in resistance to freeze/thawing stress [13].

During growth in glucose, cryoprotectanct glycerol is synthesized by a short branch of glycolysis, which involves two steps [14], [15], [16]. *Saccharomyces* yeasts have two isoenzymes for each step: GPD for glycerol-3-phosphate dehydrogenases (Gpd1p and Gpd2p) and GPP for glycerol-3-phosphatases (Gpp1p/Rhr1p and Gpp2p/Hor2p). Metabolic control analysis values calculated by flux modeling of glycerol synthesis indicate that the glycerol-3-phosphate dehydrogenase-catalyzed reaction has a flux control coefficient of approximately 0.85 and exercises the majority of flux control through this pathway in *S. cerevisiae* [17]. Moreover, *GPD1* gene overexpression increases the glycerol levels produced while the overexpression of the other three enzymes does not [16], [17], [18] whereas reduction of *GPD1* leads to a reduced flux towards glycerol [18], [19]. *GPD1* and *GPP2* genes are is essential for growth under osmotic stress and their expression is regulated by the high-osmolarity glycerol response pathway [20], whereas *GPD2* and *GPP1* are activated to equilibrate the redox balance by regenerating NADH associated with biomass production [14]. Furthermore, *GPD1* is activated in response to cold stress [12].

The study presented here looks into different regulatory mechanisms of glycerol synthesis in *S. kudriavzevii*. We observed that an increased accumulation during low temperature micro-vinifications is present in many *S. kudriavzevii* strains isolated from different regions. An effort to understand this difference at the molecular level, as compared to *S. cerevisiae*, revealed increased *GPD1* gene expression levels in *S kudriavzevii* during alcoholic fermentation and a different expression pattern for the *GPD2* gene. Furthermore, we observed increased activity and suggest that it can be explained due to increased *V*_max_ of the Gpd1p enzyme, which also explain the increased amounts of glycerol produced by *S. kudriavzevii*. Finally, we evaluated the glycerol accumulation with *S. cerevisiae, S. kudriavzevii* or a recombinant Gpd1p variant in the same background and observed that the *S. kudriavzevii* enzyme produced increased glycerol levels at 12 or 28°C.

## Materials and Methods

### Yeast Strains and Growth Conditions

Yeast strains origin, availability and other details are described in Table 1. *Saccharomyces cerevisiae* strain T73 and EC1118 were used as a wine yeast model [21], [22]. Fermol Cryophile (FCry) is a *S. cerevisiae* commercial wine yeast (AEB Group) isolated from wine fermentations, selected as a high glycerol producing strain adapted to low temperature conditions. Diploid strain BY4743 was used as a *S. cerevisiae* laboratory strain in certain experiments due to its similarity to natural diploid isolates. Type strain IFO1802 was used as the *S. kudriavzevii* representative strain. ZP591, ZP594 and ZP629 were isolated in Portugal [5] whereas CR85, CR89, CR90 and CA111 are natural *S. kudriavzevii* strains isolated in Spain [4]. Yeast cells were maintained and grown in YPD medium (2% glucose, 2% Bacto peptone and 1% yeast extract) or SC-Ura medium (YNB 6.7%, glucose 2%, Drop-out –Ura 1.92 g/L (Formedium)) at 28°C.

**Table 1:** Strains used in this study.

Strain	Species	Description
T73^1^	*S. cerevisiae*	Wine strain, Alicante, Spain (21, 22)
FCry	*S. cerevisiae*	Wine strain, commercial (AEB, France)
EC1118	*S. cerevisiae*	Wine strain, commercial (Lalvin, Canada)
ZP591	*S. kudriavzevii*	Wild strain, Portugal (5)
ZP594	*S. kudriavzevii*	Wild strain, Portugal (5)
ZP629	*S. kudriavzevii*	Wild strain, Portugal (5)
CR85	*S. kudriavzevii*	Wild strain, Spain (4)
CR859	*S. kudriavzevii*	Wild strain, Spain (4)
CR90	*S. kudriavzevii*	Wild strain, Spain (4)
CA111	*S. kudriavzevii*	Wild strain, Spain (4)
IFO1802^2^	*S. kudriavzevii*	Type strain, NCBI
BY4743^3^	*S. cerevisiae*	MATa/α his3Δ1/his3Δ1 leu2Δ0/leu2Δ0 LYS2/lys2Δ0 met15Δ0/MET15 ura3Δ0/ura3Δ0 (EUROSCARF)
BY4743gpd1Δ	*S. cerevisiae*	MATa/α his3Δ1/his3Δ1 leu2Δ0/leu2Δ0 LYS2/lys2Δ0 met15Δ0/MET15 ura3Δ0/ura3Δ0 gpd1Δ/gpd1Δ (EUROSCARF)
BY4741^3^	*S. cerevisiae*	MATa his3Δ1 leu2Δ0 lys2Δ0 met15Δ0/ura3Δ0 (EUROSCARF)
BY4741gpd1Δ	*S. cerevisiae*	MATa his3Δ1 leu2Δ0 lys2Δ0 met15Δ0 ura3Δ0 gpd1Δ (EUROSCARF)
BYpYES*GPD1*-Sc	*S. cerevisiae*	BY4741gpd1Δ pYES-*GPD1*_Scer_ (This work)
BYpYES*GPD1*-Sk	*S. cerevisiae*	BY4741gpd1Δ pYES-*GPD1*_Skud_ (This work)
BYp	*S. cerevisiae*	BY4741gpd1Δ pGREG526 (This work)
BYp*GPD1*_Scer_	*S. cerevisiae*	BY4741gpd1Δ pGREG526 -*GPD1*_Scer_ (This work)
BYp*GPD1*_Skud_	*S. cerevisiae*	BY4741gpd1Δ pGREG526 -*GPD1*_Skud_ (This work)
BYp*GPD1*_Sce-Skud_	*S. cerevisiae*	BY4741gpd1Δ pGREG526 -*GPD1*_Scer-Skud_ (This work)

Some strains are available from collections 1: CECT; 2: NBRC; 3: ATCC.

Micro-vinifications were performed with natural Bobal variety must or in MS300 synthetic media simulating standard grape juice [23]. Overnight precultures were inoculated at 5.0×10^5^ cells/ml density in 100 ml bottles with gas interchange filled with MS300. Batch fermentations were performed at 12°C with gentle agitation (120 rpm) in triplicate.

For stress experiments, yeast were grown overnight in YPD media at 28°C. Cells were diluted to OD_600_ = 0.2 and cultured at 28°C until OD_600_ = 1. Then, cells were transferred to 1 M sorbitol YPD or to 12°C pre-cold YPD. For anaerobic conditions, cells were injected into bottles without O_2_ (N_2_ bubbled until saturation). Experiments were performed in triplicate. To test glycerol production of BYp, BYp*GPD1*_ Scer,_ BYp*GPD1*_ Skud_ and BYp*GPD1*_Sce_-_Skud_ strains, the media used was SC-Ura with 10% of glucose.

### Plasmid Construction, *GPD1* Sequencing and Structure Modeling

Plasmids expressing the *S. cerevisiae* or *S. kudriavzevii GPD1* gene under GAL promoter were constructed using pYES2.1 TOPO® TA Expression Kit (Invitrogen) following manufactures’ instructions. Plasmids expressing the *S. cerevisiae* or *S. kudriavzevii GPD1* gene under its own promoter were constructed using pGREG526 by homologous recombination in yeast [24]. To construct a version with *GDP1* promoter from *S. cerevisiae* and a recombinant *GPD1* coding sequence from *S. cerevisiae* and *S. kudriavzevii* (pGREG526-*GPD1*_Scer-Skud_), the plasmid pGREG526-*GPD1*_Scer_, linearized with *Xho*I and *Aat*II, was co-transformed with a PCR product containing *GPD1*_Skud_. All constructions were confirmed by sequencing. The primers used are described in Table 2. To study gene diversity, *S. cerevisiae* and *S. kudriavzevii* IFO1802 sequences were obtained from Saccharomyces Genome Database [25] and *S. kudriavzevii* ZP591 sequence was obtained from http://www.saccharomycessensustricto.org [26]. Gpd1p enzyme structure models were built using MODWED online server based on Modeller software [27]. Three individual statistical scores (e-value, z-Dope and GA341) where used to check the model quality and the models of the two species where considered reliable. Structures were visualized with Pymol viewer [28].

**Table 2:** Primers used in this study.

Name	Sequence	Purpose	Species
GPD1-F	TGTGGTGCTTTGAAGAACG	qPCR andsequencing	*S. cerevisiae* and *S. kudriavzevii*
GPD1-R	GTTTCTTCTCTAGATTCTGG	qPCR andsequencing	*S. cerevisiae* and *S. kudriavzevii*
GPD2-F	GTTCCACAGACCWTACTTCC	qPCR	*S. cerevisiae* and *S. kudriavzevii*
GPD2-R	CCATCCCATACCTTCTACG	qPCR	*S. cerevisiae* and *S. kudriavzevii*
RHR2-F	CTTTCGATTTGGACTTCTTG	qPCR	*S. cerevisiae* and *S. kudriavzevii*
RHR2-R	GATTCGTGGTTCTTGACAAT	qPCR	*S. cerevisiae* and *S. kudriavzevii*
HOR2-F	YGCTCCAGCWGGTATTGC	qPCR	*S. cerevisiae* and *S. kudriavzevii*
HOR2-R	CRACTTCRTCTGTTTCGGC	qPCR	*S. cerevisiae* and *S. kudriavzevii*
GPD1cl-C-F	ATGTCTGCTGCTGCTGATAG	Cloning pYES2.1 TOPO	*S. cerevisiae*
GPD1cl-C-R	CTAATCTTCATGTAGATCTAA	Cloning pYES2.1 TOPO	*S. cerevisiae*
GPD1cl-K-F	ATGTCTGCTGCTGCTGATAG	Cloning pYES2.1 TOPO	*S. kudriavzevii*
GPD1cl-K-R	CTAATCTTCGTGTAGATCTAG	Cloning pYES2.1 TOPO	*S. kudriavzevii*
GPD1sq-K-F	TCCGTTATAAGTTATTCTCACC	Sequencing	*S. kudriavzevii*
GPD1sq-K-R	GCGCAAGAGCACGAGTTAAAC	Sequencing	*S. kudriavzevii*
F-ProGPD1sc	CCTAGTACGGATTAGAAGCCGCCGAGCGGGTGACATTCGATTCCGGACTCGTCC	Cloning pGREG526	*S. cerevisiae*
R-EndGPD1sc	GCGTGACATAACTAATTACATGACTCGAGGTCGACTGCGGAAGAGGTGTACAGC	Cloning pGREG526	*S. cerevisiae*
F-ProGPD1kd	CCTAGTACGGATTAGAAGCCGCCGAGCGGGTGACAGGTTCGATTCCGGACTCG	Cloning pGREG526	*S. kudriavzevii*
R-EndGPD1kd	GCGTGACATAACTAATTACATGACTCGAGGTCGACACATCGCGCAAGAGCACG	Cloning pGREG526	*S. kudriavzevii*
F-OrfGPD1kd	CCCCCTCCACAAACACAAATATTGATAATATAAAGATGTCTGCTGCTGCTGATAG	Cloning pGREG526	*S. kudriavzevii*
R-XhoGPD1kd	TCGGTTAGAGCGGATGTGG	Cloning pGREG526	*S. kudriavzevii*

### Analytical Determinations

Glycerol and sugar contents (glucose and fructose) in must samples were determined enzymatically using a commercial kit (AMS-SYSTEA) adapted to an automated ECHO instrument (Logotech), following the manufacturer’s instructions. To determine intracellular glycerol content, overnight grown YPD yeast cells were diluted to OD_600_ = 0.2 and cultured at 28°C until OD_600_ = 1. Then 10 OD_600_ units were harvested by filtration and quickly washed with 5 mL of water and transferred to a tube containing 1 ml of cold water. The yeast suspension was boiled for 10 min, cooled on ice, and centrifuged at 15,300×*g* for 10 min (4°C). The supernatant was collected and used for further analysis. Glycerol was determined using an automated ECHO instrument or by HPLC in the experiment with SC-Ura 10% Glu. A second sample (10 OD_600_ units) was harvested by filtration and left in the oven at 80°C for 24 h to determine dry weight. The values obtained are expressed as µg of glycerol per mg of yeast cells, dry weight. To measure extracellular glycerol in synthetic media 1 ml samples were centrifuged and filtered supernatant was analyzed by HPLC. Experiments were performed in triplicate.

### Gene Expression Determination

Frozen cells were lysed and homogenized by vortexing in LETS buffer (10 mm Tris pH 7.4, 10 mM lithium-EDTA, 100 mM lithium chloride, 1% lithium lauryl sulfate) with acid-washed glass beads (0.4–0.6 mm; Sigma-Aldrich) for 30 sec six times alternating with ice incubation. Total RNA was extracted using the phenol:chloroform method. Purified RNA was converted to cDNA and the expression of *GPD1*, *GPD2*, *GPP1* and *GPP2* genes was quantified by qRT-PCR (quantitative real-time PCR). Primers were designed to amplify the genes from both species. 1 mg of RNA was mixed with 0.5 mM dNTP’s, 50 pmol Oligo(dT) in 10 ml. The mixture was heated to 65°C for 5 min and quenched on ice. 10 mM dithiothreitol (DTT), 50 U of RNase inhibitor (Invitrogen) and 1x First Strand Buffer (Invitrogen) and water to 20 ml were added to the mixture, which was incubated at room temp for 2 min. After adding 200 U Superscript III (Invitrogen), samples were incubated at 42°C for 50 min and the reaction was inactivated after 15 min at 70°C. Agarose gel electrophoresis was used to check for genomic DNA contamination. qRT-PCR was performed with gene-specific primers (200 nM) (Table 2) in a 20 µl reaction, using the Light Cycler FastStart DNA MasterPLUS SYBR green (Roche Applied Science, Germany) in a LightCycler® 2.0 System (Roche Applied Science, Germany). All samples were processed for melting curve analysis, amplification efficiency and DNA concentration determination. A mixture of all samples and serial dilutions (10^−1^ to 10^−5^) was used as standard curve. The constitutive *ACT1* gene expression was used to normalize the amount of mRNA and absolute values are represented.

### Enzyme Activity Measurements

Cytoplasmic Gpd1p activity in crude extracts was assayed as described previously [14] with minimal modifications. Samples were harvested by centrifugation, washed twice with cold isosmotic media and concentrated in 0.1 M potassium phosphate buffer (pH 7.5) containing 2 mM MgCl_2_, 1 mM DTT and 1 mM EDTA. Cells were disrupted in FastPrep (MP Biomedicals) device at 4.5 m/s with glass beads in 30 s intervals over a total period of 2 min and disruption was confirmed by microscopy. Unbroken cells and debris were removed by centrifugation for 10 min at 10000 g. The supernatant was immediately used to assay Gpd1p activity. The enzymatic reaction (2 ml) contains: 20 mM imidazol–HCl (pH 7.0), 1 mM MgCl_2_, 1 mM DTT, 0.67 mM dihydroxyacetone phosphate (DHAP) and 0.09 mM NADH. One unit (U) is defined as the amount of enzyme catalyzing the conversion of 1.0 µmol of DHAP to glycerol-3-phosphate per minute at 25°C. Specific activity is expressed as units per mg of protein (U•mg^−1^). Total protein content was estimated by the Bio-Rad Protein Assay with bovine serum albumin as a standard. To determine the kinetic parameters, DHAP and NADH varied within the concentration range 0.2–4 mM and 0.02–2 mM, respectively. Activity measurements obtained with the different substrate concentrations were represented and non-linear regression was adjusted to Michaelis-Menten equation using GraphPad Prism 6.0 Software Enzyme Kinetics package, which directly calculates *V*_max_ and *K*_m_. Experiments were performed in triplicate.

## Results

### Increased Extracellular Glycerol Accumulation during Low Temperature Micro-vinifications

**Figure 1:**
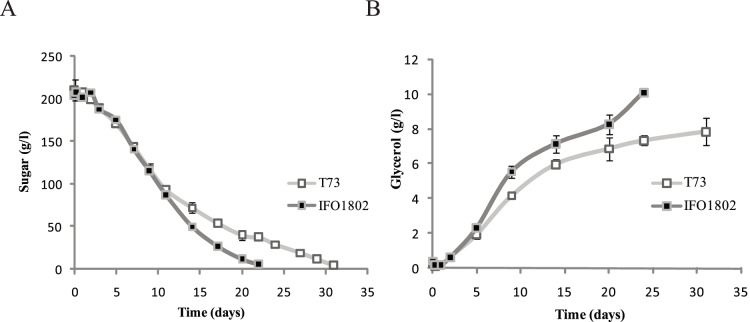
Micro-vinification experiments in natural must at low temperature with *S. cerevisiae* (T73) and *S. kudriavzevii* species (IFO1802). Precultured cells were inoculated in Bobal natural must at 12°C and samples were taken along the fermentation to determine sugars (glucose and fructose) (A) or glycerol (B) content for each species. Three independent micro-vinification bottles were used for each strain and average ± standard deviation is shown.

Micro-vinification experiments in Bobal natural must were performed with the *S. cerevisiae* wine strain T73 and with the *S. kudriavzevii* type strain IFO1802 at 12°C and fermentation performance was monitored by glucose consumption. Must samples were taken throughout the micro-vinification experiments and were used to measure extracellular sugars (Figure 1A) and extracellular glycerol concentrations (Figure 1B). As shown in Figure 1, the *S. kudriavzevii* IFO1802 strain was able to complete must fermentation at low temperature 9 days faster than the reference wine strain T73 (Figure 1A), revealing the better adaptation of *S. kudriavzevii* to cold environments. Figure 1B also shows extracellular glycerol content measured throughout the winemaking process. For both strains, the extracellular glycerol accumulation pattern can be divided into two phases: a first step of high production that ends in the interval of 5–15 days and a second period of moderate extracellular glycerol accumulation lasting until the end of the process (Figure 1B). A clear difference between both strains can be observed after 5 days, indicating IFO1802 tends to produce higher extracellular glycerol amounts than T73 strain (Figure 1B). It is interesting to note that IFO1802 strain exhibits a higher extracellular glycerol accumulation than *S. cerevisiae* T73, even when glucose consumption is very similar (between days 5 and 10 of fermentation) (Figure 1A). At the end of the micro-vinification, IFO1802 accumulated 10.1 g/l of extracellular glycerol whilst T73 only reached 7.4 g/l.

**Figure 2:**
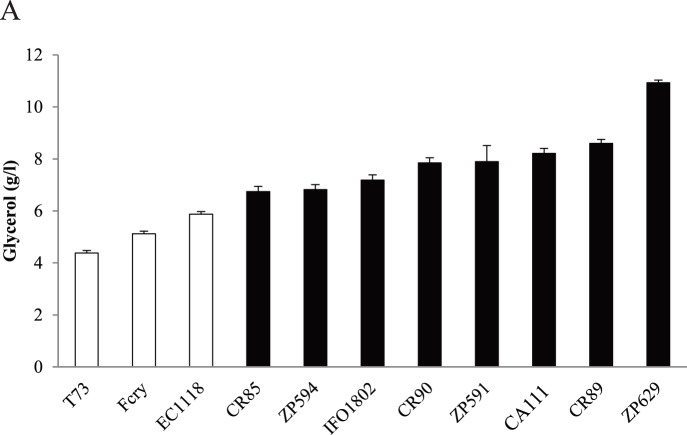
Micro-vinification experiments in synthetic must at low temperature with *S. kudriavzevii* (black bars) and *S. cerevisiae* (white bars) strains. Eight strains of *S. kudriavzevii* isolated in Japan (IFO1802), Portugal (ZP591, ZP594, ZP629) and Spain (CR85, CR89, CR90, CR111) were selected to compare with three *S. cerevisiae* (white bars) strains. Precultured cells were inoculated in synthetic must at 12°C and samples were taken after sugar exhaustion to determine glycerol content for each species. Three independent micro-vinification bottles were used for each strain and average ± standard deviation is shown.

To elucidate whether this difference was species specific or if it was only due to strain variability, micro-vinification experiments were carried out in synthetic must at 12°C with several strains of both species. The amounts of accumulated extracellular glycerol are presented in Figure 2. FCry and EC1118, two commercial wine strains showing good performance in low temperature fermentations were selected as representative of *S. cerevisiae* wine strains. Moreover, seven additional *S. kudriavzevii* strains were assayed, since this species has been less studied in fermentations than *S. cerevisiae*. Normally dry wines produced by *S. cerevisiae* contain about 5 g/l of glycerol [29] and metabolic studies have observed decreases in extracellular glycerol production as temperature drops below 26°C [8]. *S. cerevisiae* strains produced low amount of extracellular glycerol, between 4.4 and 5.8 g/l, in concordance to published levels [29] whereas *S. kudriavzevii* strains produced high levels of extracellular glycerol, between 7.0 and 10.9 g/l. The T73 strain produced the lowest amount of extracellular glycerol (4.4±0.1) while *S. kudriavzevii* ZP629 presented the highest value (10.9±0.6).

To study the relation between the increased glycerol accumulation observed in *S. kudriavzevii* strains and yeast cell resistance to osmotic stress we performed a drop test for two *S. cerevisiae* strains (T73 and EC1118) and four *S. kudriavzevii* strains (CA111, CR85, CR89 and ZP629) either at 28 or 12°C in several osmotic stress conditions (sorbitol 1.5 and 1.8 M, KCl 1.25 M and NaCl 1.0 M). The results (figure S1) suggest that increased extracellular glycerol levels do not necessarily mean increased osmotolerance since strains CR89 and especially strain CA111, that present high levels of glycerol accumulation (Figure 2), showed lower osmotic stress resistance than *S. cerevisiae* strains, especially in KCl 1.25 M and in Sorbitol 1.8 M at 28°C. On the other hand, ZP629, the strain with the highest glycerol production, showed the highest osmotolerance in most of the conditions tested (figure S1). Combination of osmotic stress and growth at 12°C produced a drastic reduction of yeast survival for all strains. In the mild osmotic stress KCl 1.25 M, only *S. kudriavzevii* strain CR85 was able to grow to some extent. Thus we can conclude that *S. kudriavzevii* strains accumulate higher amounts of glycerol that *S. cerevisiae* strains but this does not necessarily increase their osmotolerance.

### Variation of Intracellular Glycerol Content with Temperature

**Figure 3:**
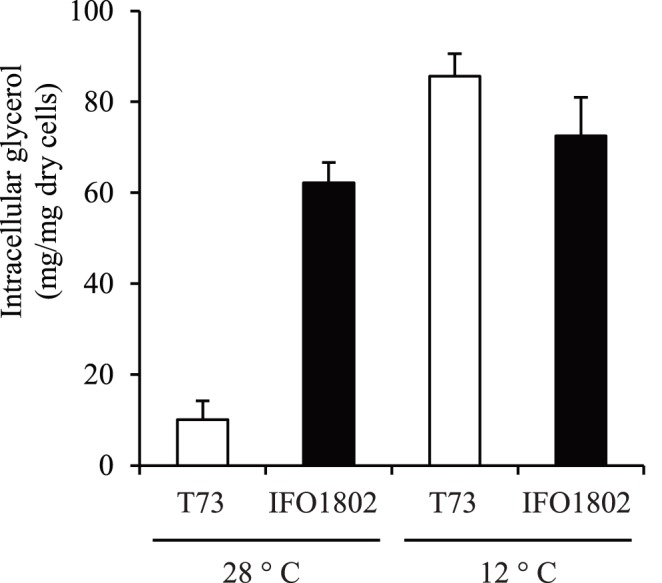
Intracellular glycerol determination for *S. kudriavzevii* (black bars) and *S. cerevisiae* (white bars) strains. A strain of *S. kudriavzevii* (IFO1802) was selected to compare with a *S. cerevisiae* (T73) strain the intracellular content of glycerol. Batch cultures at 12 or 28°C in YPD medium were performed until OD_600_ = 1. Then, cells were recovered by filtration, washed and glycerol was measured in the cell extracts. Three independent batches were used for each strain and averages ± standard deviation are normalized against *S. cerevisiae* value and expressed as µg of glycerol per mg of yeast cells, dry weight.

To determine whether the higher amounts of extracellular glycerol produced by *S. kudriavzevii* strains reflect an increase in the production of this metabolite inside the cells in standard growth conditions, we measured the intracellular glycerol content in batch cultures at 28 or 12°C in YPD at OD_600_ = 1 (Figure 3). The IFO1802 intracellular glycerol content was 6.2 times higher than T73 strain at 28°C. However no significant differences were observed at 12°C between the two strains. *S. kudriavzevii* maintain elevated intracellular glycerol levels at both temperatures whereas *S. cerevisiae* strain is able to increase intracellular glycerol content in response to cold conditions as was previously described [12].

### Gene Expression of Glycerol Synthesis Related Genes

**Figure 4:**
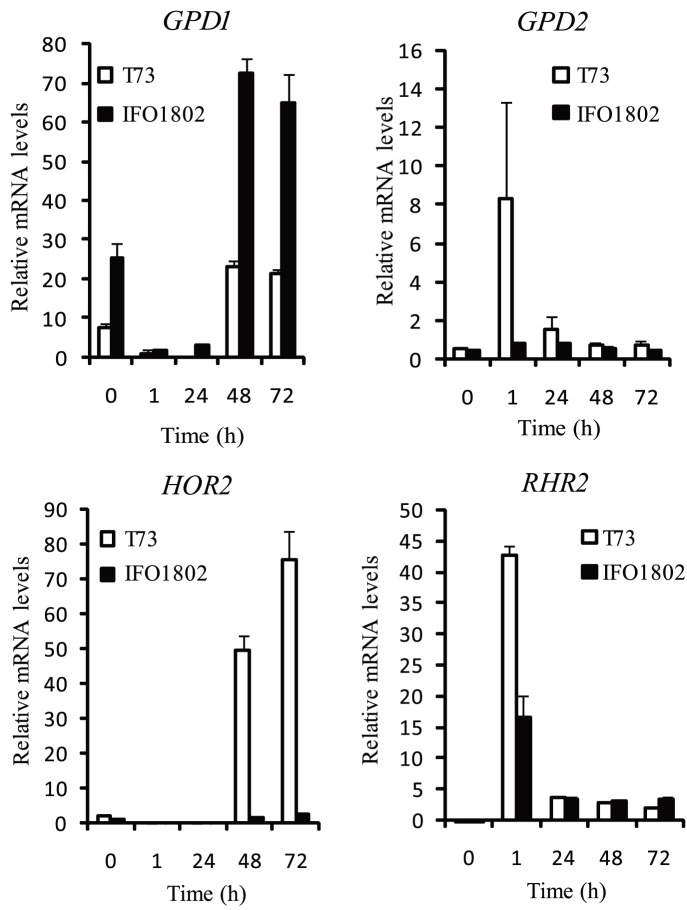
Expression of glycerol biosynthetic genes during first hours of low temperature micro-vinifications in synthetic must for *S. kudriavzevii* (black bars) and *S. cerevisiae* (white bars). *S. kudriavzevii* (IFO1802) was compared to *S. cerevisiae* (T73) in genes responsible for the first (*GPD1*, *GPD2*) and second (*GPP1, GPP2*) steps of specific glycerol biosynthetic pathway. Samples were taken in the first part of synthetic must micro-vinifications at 12°C. After RNA extraction, expression of the different genes was determined by qPCR and values were normalised with *ACT1* constitutive gene and absolute levels are shown. Three independent micro-vinification bottles were used for each strain and averages ± standard deviation are shown.

In order to test whether glycerol accumulation in *S. kudriavzevii* was related to gene expression, we studied genes related to glycerol synthesis by the qPCR technique during the first days of synthetic must fermentation at 12°C. We focused our analysis on the two genes encoding glycerol-3-phosphate dehydrogenase (GPDH) isoforms *GPD1* and *GPD2,* and in the two genes encoding glycerol-3-phosphatase (GPP) isoforms: *GPP1*/*RHR2* and *GPP2*/*HOR2*. As can be seen in Figure 4, *GPD2, GPP2* and *GPP1* presented similar or reduced mRNA levels in some time points in strain IFO1802 compared to T73. In contrast, the expression of *GPD1* exhibited an increased level (between 3.1–3.8 fold at 0, 48 or 72 h time points) in the *S. kudriavzevii* strain IFO1820 (Figure 4).

**Figure 5:**
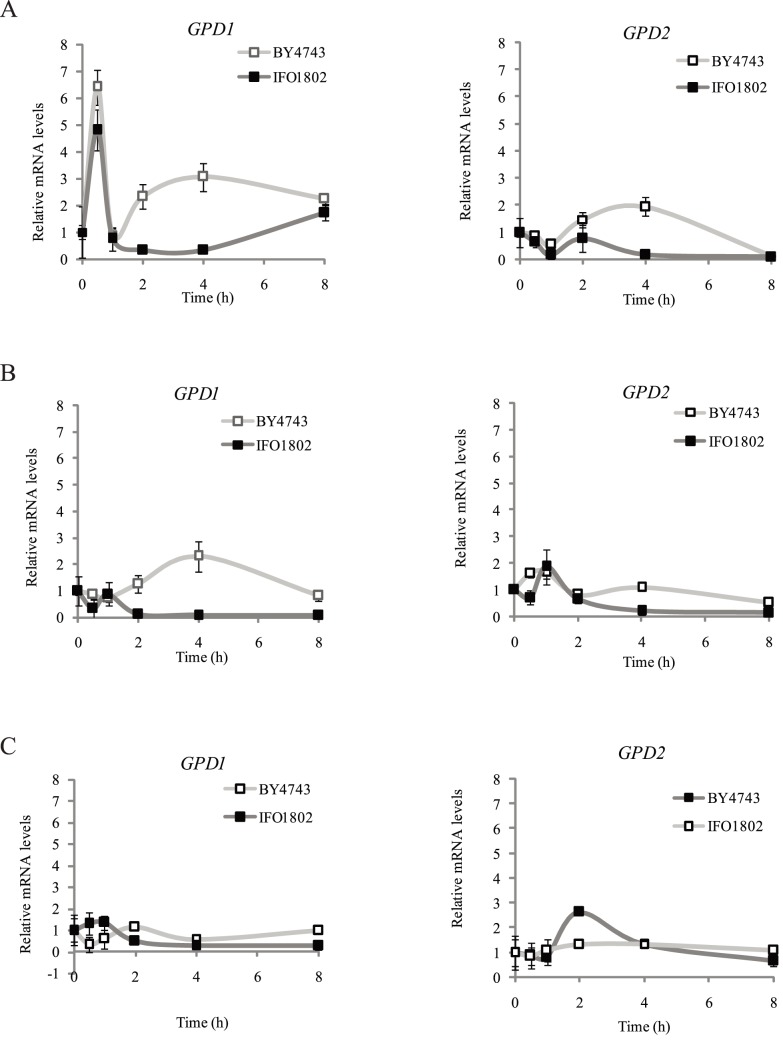
Expression of glycerol biosynthetic genes after osmotic (A), cold (B), and anaerobic stress (C) in laboratory conditions for *S. kudriavzevii* (black squares) and *S. cerevisiae* (white squares). A strain of *S. kudriavzevii* (IFO1802) was selected to compare with a *S. cerevisiae* (BY4743) strain the expression of the genes responsible for the first (*GPD1*, *GPD2*) steps of specific glycerol biosynthetic pathway. Batch cultures at 28°C in YPD medium were grown until OD_600_ = 1. Then, cells were transferred to 1 M sorbitol YPD (A), to 12°C pre-cold YPD (B) or to YPD in bottles without O_2_ (N_2_ bubbled until saturation) (C) and samples were taken after 0, 0.5, 1, 2, 4 and 8 h. After RNA extraction, expression of the different genes was determined by qPCR and values were normalised with *ACT1* constitutive gene and relativized to time point 0 h. Three independent flask or bottles were used for each strain and averages ± standard deviation are shown.

In the micro-vinification experiments, cells are subjected to anaerobiosis and different stress conditions, mainly osmotic and cold stress [29]. All these conditions have been described to increase *GPD1* expression in *S. cerevisiae* [12], [13], [14], [20]. To study *S. kudriavzevii GPD1* activation in response to classical stress induction experiments were performed to compare with *S. cerevisiae*. The *S. cerevisiae* laboratory diploid strain BY4743 background was used to take advantage of the deletion mutant collection and because it has been used as a laboratory model strain in many studies [12]. We performed batch cultures with BY4743 and IFO1802 strains and subjected the cells to osmotic stress (Figure 5A), cold stress (Figure 5B) or anaerobiosis (Figure 5C) in standard laboratory conditions. The results shown in Figure 5 indicated an increase in *GPD1* gene expression in response to osmotic (Figure 5A) and cold stress (Figure 5B) in the *S. cerevisiae* strain, as described previously [12], [14]. The *S. kudriavzevii* strain presented also an early activated expression (0–1 h) in response to osmotic stress but levels were decreased in the later time points (2–8 h) (Figure 5A). IFO1802 *GPD1* gene also showed lower mRNA levels in response to cold (Figure 5B) or anaerobic stress (Figure 5C) compared to *S. cerevisiae*. *GPD2* gene expression levels in response to anaerobiosis were also tested (Figure 5C). *S. cerevisiae* showed higher levels in response to anaerobiosis whereas *S. kudriavzevii* strain showed higher *GPD2* mRNA levels in osmotic stress compared to *S. cerevisiae*. All these data suggest that glycerol synthesis related genes are regulated differently in *S. cerevisiae* and *S. kudriavzevii.*

### Increased GPDH Activity in *S. kudriavzevii*

**Figure 6:**
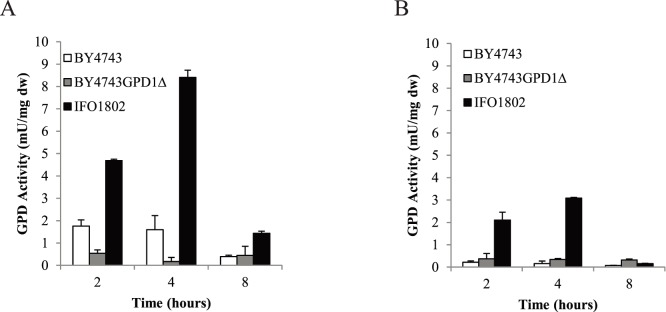
Determination of glycerol-3-phosphate 1 activity after osmotic (A) and cold stress (B) in laboratory conditions for *S. kudriavzevii* (black bars) and *S. cerevisiae* (white bars). A strain of *S. kudriavzevii* (IFO1802) was selected to compare with a *S. cerevisiae* (BY4743) strain the Gpd1p activity. A *S. cerevisiae* strain without *GPD1* gene (BY4743GPD1Δ, grey bars) was included to evaluate Gpd2p contribution to total activity. Batch cultures at 28°C in YPD medium were performed until OD_600_ = 1. Then, cells were transferred to 1 M sorbitol YPD YPD (A) or 12°C pre-cold (B) and samples were taken after 2, 4 and 8 h. Gpd1p activity was determined in the cell extract and values were normalised with total protein content. Specific activity is expressed as miliunits per mg of proteins (mU⋅mg^−1^). Three independent batches were used for each strain were performed in triplicate and averages ± standard deviation are shown.

GPDH activity has been well correlated with glycerol production since this enzyme has a flux control coefficient of approximately 0.85 [30]. Since higher glycerol production could be the result of increased activity of either isoform [31], we were also interested in testing GPDH activity in response to specific stresses for both species. The results in Figure 6 show that IFO1802 exhibits significantly higher GPDH activity after osmotic (2.6, 5.2 and 3.6 fold after 2, 4 and 8 hour respectively) (Figure 6A) and cold stresses (9.7, 19.9 and 2.2 fold after 2, 4 and 8 hour respectively) (Figure 6B). It is worth noting that increased GPDH activity is observed in *S. kudriavzevii* in cold stress (Figure 6), whereas no *GPD1* mRNA increase was detected (Figure 5B). To check Gpd2p contribution to the GPDH activity determination, we performed the same experiment with the BY4743gpd1Δ strain and no significant differences (p<0.05) were observed comparing to wild type strain after 2 or 4 h, although a possible contribution of Gpd2p cannot be completely discarded, especially after 8 h after osmotic stress and in cold stress samples. Increased GPDH activity in *S. kudriavzevii* can be a consequence of increased content of the Gpd1 protein or it can be due to enhanced kinetic properties of Gpd1p enzyme. New experiments were performed to explore this later possibility.

### Gpd1p Sequence and Structure Modeling

To test if the differences in Gpd1p activity between the two species were somehow related to differences in the protein sequence or structure we compared Gpd1p sequences of 24 *S. cerevisiae* strains obtained from the SGD database and three *S. kudriavzevii* strains, two obtained from the database (IFO1802 and ZP591) and one sequence obtained by us (Gene Bank accession number KF700356) from strain CR89. Intraspecific changes were not observed; however Gpd1p from *S. kudriavzevii* presented five conserved amino acid replacements compared to *S. cerevisiae* (Ala31Ile, Ile67leu, Glu76Asp, Asp142Asn and Ser143Pro) out of 391 total residues, corresponding to an identity of 98.7% (figure S2). Two of these changes (Glu76Asp and Ser143Pro) were also observed in *S. bayanus*. This elevated identity was expected since this enzyme is highly conserved among yeast strains and even within eukaryotes. To determine whether any of the five changes affected the tertiary structure of the Gpd1p enzyme, we performed structure modeling and we compared the enzymes from the two species (figure S3A). Although both Gpd1p versions adopt very similar conformations, maintaining main secondary structures, several residues involved in the catalytic active site (Arg310, Asn246, Thr305, Lys245, Lys152, Asp205) showed positional differences ranging from 1.0 to 2.4 Å (figure S3B). This study suggested that, among the five residues that are different between *S. cerevisiae* and *S. kudriavzevii*, the two residues that can have more influence in the catalytic properties are in positions 142 and 143, which are close to other residues involved in NAD binding (Phe129 and Lys152) (figure S3C).

### Gpd1p from *S. kudriavzevii* Exhibits a Higher V_max_

We studied whether the differences in glycerol accumulation observed between *S. kudriavzevii* and *S. cerevisiae* could be explained by differences in the catalytic properties of the Gpd1p enzyme. To that end, kinetic assays were performed to determine *K*_m_ and V_max_ of *S. cerevisiae* and *S. kudriavzevii* Gpd1p enzymes. The *K*_m_ and V_max_ for the two substrates, dihydroxyacetone phosphate (DHAP) and NADH, was performed. The assay provided reproducible and consistent data, since values are within the range previously described for the purified enzyme [14]. To perform the experiment BY4741gpd1Δ strain was complemented with multicopy expression plasmids containing (pYES) either the *S. cerevisiae* or *S. kudriavzevii GPD1* genes under the strong promoter pGAL1. Overnight precultures were used to inoculate exponential cultures in YPD galactose medium. As a control we checked that no GPD activity was observed in the BY4741gpd1Δ strain without plasmid in the induction conditions. *V*_max_ and *K*_m_ values determined for both Gpd1p substrates were calculated (Table 3). *V*_max_^DHAP^ determinations revealed significant differences between the two species whereas *K*_m_^NADH^, *K*_m_^DHAP^ and *V*_max_^NADH^ did not differ significantly. The higher catalytic rate of *S. kudriavzevii* Gpd1p, together with the overexpression of its encoding gene, may explain that *S. kudriavzevii* has a generally higher production of glycerol than *S. cerevisiae*. Taking into account that Gpd1p is the flux controlling enzyme in the pathway, the effect of increased *V*_max_^DHAP^ on the glycerol accumulation could be highly significant, even if the increase is only around 20%.

**Table 3:** Gpd1p kinetic parameters.

	DHAP		NADH	
	*K*_m_	V_max_	*K*_m_	V_max_
BY4741-*GPD1*- *S. cerevisiae*	0,54±0,06	87,34±4,5	0,051±0,02	48,85±3,9
BY4741-*GPD1*- *S. kudriavzevii*	0,61±0,05	108,0±4,1*	0,11±0,02*	58,91±3,8

* p<0.05 significant differences *S. cerevisiae* versus *S. kudriavzevii.*

### *Gpd1p* from *S. kudriavzevii* Produces more Extracellular Glycerol

**Figure 7:**
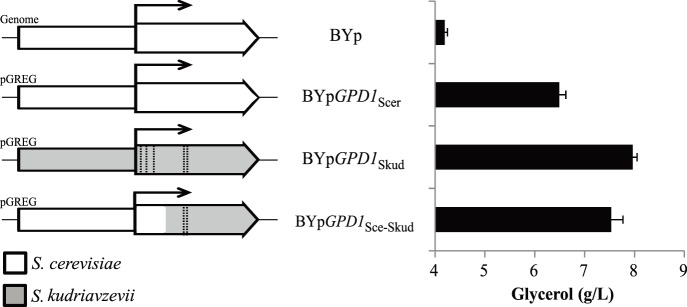
Glycerol production in strains with different versions of *GPD1*. Strains with wild type *GPD1* (BYp) or plasmid containing *GPD1* from *S. cerevisiae* (BYp*GPD1*_Scer_), *S. kudriavzevii* (BYp*GPD1*_Skud_) or a recombinant *S. cerevisiae* - *S. kudriavzevii* coding sequence (BYp*GPD1*_Sce-Skud_), were grown in SC-Ura 10% glucose media until sugar exhaustion at 28 or 12°C. Aminoacidic changes observed in the *S. kudriavzevii* enzyme are represented as vertical black lines. Extracellular metabolites were determined with HPLC method. Biological triplicates were performed and averages ± standard deviation are shown.

In order to evaluate the metabolic effect of the presence of a *S. cerevisiae* or *S. kudriavzevii* Gpd1p enzyme, we expressed, with the low copy plasmid pGREG526, the *GPD1* gene of *S. cerevisiae* (BYp*GPD1*_Scer_) or *S. kudriavzevii* (BYp*GPD1*_Skud_) under their own promoter in the background strain BY4741gpd1Δ. These strains and the BY4741 wild type containing the empty vector (BYp), were inoculated in selective minimal media with 10% glucose and the glycerol was measured after sugar exhaustion by HPLC. The experiment was performed at 12 and 28°C. The results (Figure 7) showed that expression of either gene increased the amount of extracellular glycerol produced at both temperatures. Interestingly, the strain containing *S. kudriavzevii* Gpd1p enzyme produced 22.8% more extracellular glycerol than the one with *S. cerevisiae* Gpd1p at 28°C and 24.9% more at 12°C (Figure 7), a significant increment comparable to the difference in *V*_max_^DHAP^ observed between both enzymes. In order to elucidate if the increased extracellular glycerol accumulation was because of the changes in two residues (142 and 143) situated in the vicinity of the NAD binding site, we constructed a strain (BYp*GPD1*_Sce_-_Skud_) containing the *S. cerevisiae GPD1* promoter next to a recombinant *S. cerevisiae*-*S. kudriavzevii GPD1* coding sequence containing the residues 142 and 143 from *S. kudriavzevii* (Figure 7) and performed the same experiment. The results (Figure 7) showed that BYp*GPD1*_Sce-Skud_ also produced a significant (p<0.001) increase in glycerol accumulation (16.0%) respect to BYp*GPD1*_Scer_ at 28°C. The glycerol produced with BYp*GPD1*_Sce-Skud_ and BYp*GPD1*_Skud_ at 28°C was not significantly different suggesting that changes in residues 142 and 143 are sufficient to explain increased glycerol accumulation at this temperature. Also, the glycerol produced with BYp*GPD1*_Sce-Skud_ at 12°C was not significantly different to BYp*GPD1*_Scer_. It is interesting to note, that no significant differences were observed in the ethanol production among the different strains (results not shown).

## Discussion

The industrial relevance of glycerol production by yeasts, especially for wine production, launched many studies that tried to increase it. Classic examples were the increase in *GPD1* expression and other genes to counteract the side-effect of higher acetate production [32], [33], [34]. This study was focused in *S. kudriavzevii* species which naturally produces increasing glycerol levels compared to *S. cerevisiae*. As our results showed, the increased extracellular glycerol synthesis at low temperatures is present in many *S. kudriavzevii* strains isolated from different regions and therefore it is a species-specific trait. The different metabolism in the two species can be explained by increased expression of metabolic genes underlying increased glycerol accumulation in *S. kudriavzevii*. In fact, we observed increased *GPD1* gene expression during must fermentation in *S. kudriavzevii* compared to *S. cerevisiae*. Unexpectedly, the genes *GPP1*, *GPP2* and *GPD2* showed decreased expression levels which may be explained by the evolution of *S. cerevisiae* to elevated levels of regulation of glycerol biosynthesis. However, we have also observed other differences at the protein level, which may contribute to the increase in glycerol production. *S. kudriavzevii* presents increased GPDH specific activity compared to *S. cerevisiae* in different laboratory conditions. Tertiary structure prediction revealed certain differences suggesting potential disparity in enzymatic activity and in kinetic properties. Taking into account the structural differences detected, we observed an increased V_max_ in *S. kudriavzevii* Gpd1 enzyme compared to *S. cerevisiae*, which can explain the increased specific activity and therefore the increased glycerol levels. Also, previous work has described increased *S. kudriavzevii* GPDH activity respect to *S. cerevisiae,* especially in cold stress conditions [8], supporting our model. Finally, direct comparison of the two *GPD1* and a recombinant version in the same background let us determine that the changes in the residues 142 and 143 of *S. kudriavzevii* have a significant impact in the increased ability of the enzyme to produce glycerol, although residues 31, 67 and 76 are relevant for low temperature glycerol production. Intracellular glycerol accumulation and Gpd1p activity was high in *S. kudriavzevii* at 12 but also at 28°C. This suggests that, as a consequence of *S. kudriavzevii* adaptation to low temperatures, this species has high flux to glycerol biosynthetic pathway even at high temperatures.

Glycerol production is a key process to resist osmotic stress in yeast cells [14], [15], [16], [20]. Some *S. kudriavzevii* strains showed a correspondence between osmotolerance and glycerol levels produced after glucose fermentation but other strains did not (figure S1), [7]. This may reflect that osmotolerance depends as well on other key players. For example, upon hyperosmotic shock, cells first mobilize a rapid rescue system that prevents excessive loss of ions and water. The potassium antiporters Nha1p and Nhx1p are implicated in response to cell shrinkage upon osmotic stress and their presence in cells is important for recovery from sudden exposure to hyperosmotic media [35], [36]. Thus, changes in efficiency of any of those players can explain why some *S. kudriavzevii* strains that show increased glycerol accumulation are not more osmotolerant than *S. cerevisiae* strains. So, new studies on the functional differences in any of those proteins will be important to understand the glycerol level differences among the two species.

We have studied the regulation of glycerol synthesis in *S. kudriavzevii* compared to *S. cerevisiae* at low temperatures. The results reveal important differences between the two species, supporting a significant disparity in the central carbon metabolism, probably due to adaptation to specific environments. In this respect, we showed in a recent work [37] that temperature has influenced the evolution of the *Saccharomyces* genus, favoring the adaptation of some species to grow at either lower (*S. kudriavzevii*) or higher (especially *S. cerevisiae*) temperatures. We hypothesized that, in a first evolutionary event, *S. cerevisiae* and other species diverged from *S. kudriavzevii* and *S. uvarum,* which are better adapted to grow at low temperatures. In fact, all these diversifications were preceded by whole genome duplication (WGD), which increased glycolytic flux [38]. They suggested that this bestows selective advantages on yeast when competing for resources to growth in conditions with high sugar levels. Notwithstanding, we hypothesize that *Saccharomyces* species developed two main strategies after the WGD to fine tune its metabolism and adapt to different niches. Some species, like *S. kudriavzevii*, derived this increased glycolytic flux towards the production of elevated levels of cryoprotectanct glycerol. This strategy has enabled them to adapt to low temperature environments and maintain the NAD^+^/NADH ratio in alcoholic fermentations. However, other species like *S. cerevisiae* took advantage of the increased glycolytic flux and promoted increased levels of ethanol production. Other authors [37] have proposed that *S. cerevisiae* followed this evolutionary strategy to better compete with other microorganisms for resources. It is reasonable to suppose that *S. cerevisiae* has developed a much tighter regulation of the glycerol biosynthetic pathway to redirect the glycolytic flux from glycerol to ethanol and maximize its production. In fact our results reveal high complexity levels in *S. cerevisiae* on the regulation of glycerol biosynthetic pathway genes. In *S. cerevisiae*, *GPD2, GPP1* and *GPP2* expression is induced during must fermentation at certain time points, whereas in *S. kudriavzevii* these genes show lower or no induction. The same occurs when we study stress response where lower or no inductions where observed for *GPD1* or *GPD2* genes.

In conclusion, the species *S. kudriavzevii* is able to produce high levels of glycerol and grow at low temperatures. The data obtained in this work place *S. kudriavzevii* adaptation mainly at the Gpd1p enzymatic level. By contrast, *S. cerevisiae* evolution is linked more closely to the increased gene expression regulation of glycerol synthetic pathway genes. The comparison of our data with data obtained for other *Saccharomyces* species will shed more light on the adaptive mechanisms of these yeasts. This work can have a relevant, practical, use, taking advantage of industrially relevant properties by using *S. kudriavzevii* or *S. cerevisiae* - *S. kudriavzevii* hybrids in wine production [1], [2], [4], [5], [6].

## Supporting Information

Figure S1 Evaluation of osmotolerance of different *S. cerevisiae* and *S. kudriavzevii* strains. After adjusting all strains to OD_600_ = 0.3 of YPD batch cultures, 6 serial dilutions (1/5) of *S. cerevisiae* (T73 and EC1118) and *S. kudriavzevii* strains (CA111, IFO1802, CR85, CR89 and ZP629) where spotted on YPD with glucose or mannitol as a carbon source and with different osmotic stressors (Sorbitol 1.5 or 1.8 M; KCl 1.25 M; NaCl 1.0 M). No image is presented in the conditions where no growth was observed. Plates were incubated at 28°C or 12°C.
(EPS)

Figure S2 Alignment of Gpd1p sequences from different *Saccharomyces* species. The sequences of *S. cerevisiae* strain S288C, *S. paradoxus* strain Y-17217, *S. bayanus* strain 623-6c and *S. kudriavzevii* strains ZP591 and CR89 were aligned using MEGA software. Amino acidic variations comparing with S288C are shown.
(EPS)

Figure S3 Comparison of *S. cerevisiae* and *S. kudriavzevii* Gpd1p three-dimensional structure models. The whole protein model is compared in panel A showing *S. cerevisiae* variant in red and *S. kudriavzevii* in green. Panel B compares side-chain differential position of Lys152, Asp205 and Arg310, three amino acids involved in the catalytic center. Panel C shows side-chains and relative position of dipeptides 142–143 respect to amino acids Phe129 and Lys152, involved in NADH binding. Models were built using MODWED online server based on Modeller software [27]. Structures were visualized with Pymol viewer [28].
(EPS)
